# Annotations

**Published:** 1910-06-25

**Authors:** 


### Annotations.

### The late Mrs. Stanley Boyd, M.D.

The London School of Medicine for Women has lost a staunch friend and supporter in Mrs. Florence Nightingale Boyd, the wife of Mr. Stanley Boyd and one of the best known woman doctors in London, whose death was announced last week. Mrs. Boyd, who had been ailing for some months, found it impossible to continue her work at the New Hospital for Women in consequence of ill-health, and accordingly resigned her appointment as senior surgeon last month. She was an old student of the school, to which she was attached all through her professional life, taking her Irish Licentiateship in medicine and surgery, and finally in 1888 graduating Doctor of Medicine at Brussels with the highest possible distinction. She then, after doing the usual house appointments, worked as clinical assistant at the East London Hospital for Women and Children, Shadwell, and was appointed surgeon to the Vigilance Medical Home for Women. Later on she joined the staff of the New Hospital as surgeon, and of the London School of Medicine for Women as lecturer, demonstrator of anatomy, and examiner in midwifery, finally becoming lecturer on gynæcology. She was vice-president and honorary treasurer of the Association of Registered Medical Women, a member of the British Medical Association, and a member of the Royal Society of Medicine. In questions concerning the higher education of women Mrs. Boyd always took a great interest, and as lecturer and surgeon she won a deserved popularity with her students.

### Sir William Macewen.

We sent a copy of our issue of the 18th inst. to Sir William Macewen, in which we published an account of certain proceedings attributed to him at the time of King Edward's death. On Friday afternoon, after the distribution of last week's issue, we received the following telegram: "Editor's letter received; statement conveys wholly false impression and should not be published.—William Macewen." Sir William, it will be observed, does not state how, why, or where the statement we published conveys a wholly false impression, and we must press Sir William Macewen for full particulars of the grounds on which he makes this claim. The facts were precisely stated. Sir William Macewen's telegram does not in any way question the accuracy of those facts, and it is due to the profession and the public as well as to Sir William Macewen's position in the Royal Household that he should explain how they convey a "wholly false impression." The *Glasgow Daily Record* has published the views of a few anonymous persons, who suggest that the bands were not sterilised and that nurses do not wear mourning bands in the operation theatre. We presume that the authorities of the Western Infirmary were fully alive to points of this kind, and have no doubt that they took the necessary precautions. Such suggestions do not justify or account for the proceedings we reported. Another statement is that "the mourning band is a military institution solely," whereas we were careful to point out that the practice for nurses to wear these bands originated with Lord Clarendon when Lord Chamberlain at the time of the death of Queen Victoria. Having regard to the fact that Sir William Macewen was a surgeon-in-ordinary to the late King Edward, and that no other surgeon has made any objection to his nurses showing proper respect for their dead King, it is our plain duty again to press Sir William Macewen for some adequate explanation of the extraordinary proceedings attributed to him. We would remind him that a statement of fact does not necessarily convey any impression, false or otherwise, when, as in the present case, it is free from comment.

### Scepticism and Dogma.

Doctors are frequently accused of being hopelessly dogmatic. Yet we doubt if the members of any other profession pass through a period of scepticism so intense and so severe as that which the young medical man has to encounter. He finds the most cherished opinions of one of his teachers scoffed at by another teacher whom he equally respects, and at the same time he sees the patients of the one do as well—or as badly—as those of the other. He reads, in the course of his career, books which tear to shreds the tenets that have been instilled into him during childhood. He sees divine justice apparently dealt out in most unequal portions, for the life of the keen, strong man who is of use to those around him is taken, and the useless wastrel passes through the most serious disorder only to be restored to a career as useless and as idle as it was before. By the time the medical student has been qualified a twelvemonth he very likely thinks that no drug in the pharmacopœia is of any value whatever, that only one or two lines of treatment are worth even attempting, and that, if these are not applicable, the patient will either recover or die in accordance with some fatalistic law the limits of which we have not as yet divined. He goes out into the world with the idea that the farce of treatment must be kept up, and soon finds that most treatment is really faith-healing. It is here that dogma begins to step in. When he orders his bottle of medicine he knows that it will do no good unless he can persuade the patient to believe in its efficacy, and such faith he cannot instil unless he himself can move mountains with it, or at least give an impression of his ability to do so. Therefore he has to state in a dogmatic form what he only half believes himself in order to persuade his patient. As time goes on the habit of dogma grows, and he now states readily what before he laboriously forced himself to say. From being a sceptic of sceptics he becomes as dogmatic as any divine. Some such process as this explains the apparent paradox that so many medical men are over-dogmatic, while all pass through a training which at some time or other must make them sceptical. No doubt there are many exceptions to this rule, and many medical men remain sceptics to the end of their days, while others hide their scepticism under the veneer of polite belief.

